# Hypotheses generation as supervised link discovery with automated class labeling on large-scale biomedical concept networks

**DOI:** 10.1186/1471-2164-13-S3-S5

**Published:** 2012-06-11

**Authors:** Jayasimha Reddy Katukuri, Ying Xie, Vijay V Raghavan, Ashish Gupta

**Affiliations:** 1 Center for Advanced Computer Studies, University of Louisiana at Lafayette, Lafayette, Louisiana, 70504, USA; 2 Department of Computer Science, Kennesaw State University, Kennesaw, Georgia, 30144, USA

## Abstract

Computational approaches to generate hypotheses from biomedical literature have been studied intensively in recent years. Nevertheless, it still remains a challenge to automatically discover novel, cross-silo biomedical hypotheses from large-scale literature repositories. In order to address this challenge, we first model a biomedical literature repository as a comprehensive network of biomedical concepts and formulate hypotheses generation as a process of link discovery on the concept network. We extract the relevant information from the biomedical literature corpus and generate a concept network and concept-author map on a cluster using Map-Reduce frame-work. We extract a set of heterogeneous features such as random walk based features, neighborhood features and common author features. The potential number of links to consider for the possibility of link discovery is large in our concept network and to address the scalability problem, the features from a concept network are extracted using a cluster with Map-Reduce framework. We further model link discovery as a classification problem carried out on a training data set automatically extracted from two network snapshots taken in two consecutive time duration. A set of heterogeneous features, which cover both topological and semantic features derived from the concept network, have been studied with respect to their impacts on the accuracy of the proposed supervised link discovery process. A case study of hypotheses generation based on the proposed method has been presented in the paper.

## Introduction

Text mining of biomedical literature is a research area that has attracted lot of attention in the last 5 to 10 years. Swanson [1] was one of the proponents of the hypotheses discovery from biomedical literature. As a result of his pioneering work in hypotheses discovery, Swanson discovered a novel connection between Raynaud's disease and fish oil by examining two disjoint biomedical literature sets [1]. The hypothesis of the beneficial effect of fish oil on Raynaud's disease was confirmed by an independent clinical trial two years later, which demonstrated the value of biomedical literature mining in scientific discovery. Swanson's hypothesizing model, the so called Swanson's ABC model, can be simply described as A relates to B, B relates to C, therefore A may relate to C, [2]. Ever since Swanson's discovery, a lot of research works have been carried out with the aim of automating and refining Swanson's ABC model [1,3-8]. Nevertheless, most of these reported approaches are based on analyzing the retrieval result set for one or two initial topics provided as query by a user, instead of being able to scale up to the whole set of literature database for the purpose of discovering real, novel and cross-silo biomedical hypotheses.

In recent years, link discovery has been extensively studied on social networks such as those obtained from Facebook data and bibliographic databases maintained by DBLP. As an important problem of link mining, link discovery refers to the discovery of future links between objects (or nodes) that are not directly connected in the current snapshot of a given network. In [9], Őzgür and his colleagues applied link discovery technique to generate hypotheses on relationships between genes and vaccines. This work first extracted networks on gene-gene interactions and gene-vaccine interactions from literature with the help of gene and vaccine ontology; then analyzed the networks by computing different types of centrality measures for each node in the networks. Given its restricted focus on gene and vaccine relationships, this work by its nature was not designed for cross-silo biomedical discovery.

In order to address the challenge of large-scale cross-silo biomedical hypotheses discovery, in this paper, we first model a biomedical literature repository as a comprehensive network of biomedical concepts belonging to different semantic types. Then we extract such a large-scale concept network form Medline [10]. We further calculate a variety of topological and semantic features from the concept network and model the hypotheses discovery as a classification problem based on those features. Moreover, in order to automatically build the classification model for prediction, we take two snapshots of the concept networks corresponding to two consecutive time durations, such that a training data set can be formed based on a group of labeled concept pairs that are automatically extracted from the snapshots. We further extract multiple heterogeneous features for labeled concept pairs solely from the first snapshot of the concept network. The impact of those heterogeneous features on hypotheses discovery has been studied.

The rest of the paper will be organized as follows. In the Related work section, we briefly describe relevant works in biomedical hypotheses discovery and link mining. In the Hypotheses generation as supervised link discovery on biomedical concept network section, we formulate hypotheses generation from literature as link discovery in a concept network and further model the link discovery as a supervised learning process based on a set of topological and semantic features. In the Concept network creation and feature extraction using Map-Reduce framework section, we address the challenges of extracting large-scale concept networks from literature corpus. We also address the challenges involved in automatically generating labeled data and extracting heterogeneous features for a large number of labeled data using Map-Reduce framework. In the Experimental results section, we present experimental results. Finally, we conclude our paper with the Conclusions section.

## Related work

Swanson's pioneering work in 1986 on biomedical hypotheses generation led to the discovery of the novel connection between Raynaud's disease and fish oil by examining two disjoint biomedical literature sets (Swanson [1]). In his follow-up work in 1990, Swanson suggested a trial-and-error search strategy, by which the ABC model guides a manual online search for identifying logically related non-interactive literature (Swanson [7]). By applying this strategy for biomedical literature analysis, Swanson discovered some other novel biomedical hypotheses, such as the implicit connection between the blood levels of Somatomedin C and dietary amino acids arginine (Swanson [7,11]), and hidden link between the mineral magnesium and treating the medical problem causing migraine headaches (Swanson [7]).

Along with the advances in the text retrieval and mining techniques, researchers have made several efforts to partially automate Swanson's ABC model for hypotheses generation. Stegmann and Grohman proposed a way to guide a researcher to identify a set of promising B terms by conducting clustering analyses of terms on both the retrieval result set of topic A and the retrieval result set of topic C (Stegman et al. [6]). Their work used measures called centrality and density to evaluate the goodness of term clusters and showed that the promising B terms that link disjoint literature for topics A and C tend to appear in clusters of low centrality and density. Srinivasan's approach to identify promising B terms starts with building two profiles for both topic A and topic C, respectively, from the retrieval result sets of A and C [5]. In her work, the profile of a topic consists of terms that have high frequency in the retrieval result set of that topic and belong to semantic types of interest to the user. Then the intersection of A's profile with C's profile generates the candidate B terms. The process of identifying B terms from given topics A and C is called closed discovery. In her work, Srinivansan also applies the topic profile idea to conduct open discovery, which identifies both B terms and C terms given only topic A. Srinivansan's open discovery algorithm can be simply described as follows: Top-ranking B terms are selected from the profile of topic A. Then, a profile for each selected B term is created from the retrieval result set of that B term. The top-ranking terms in a B term's profile form candidate C terms. If topic A's retrieval result set is disjoint from a candidate C term's retrieval result set, then this candidate C term is reported as having potential relationship with topic A via term B. Slightly different from Srinivansan's topic profile approach, Pratt and Yildiz directly applied association mining on the retrieval result set of topic A to conduct open discovery [4]. In their work, the logical inference based on two association rules A→B, B→C leads to the finding of a candidate C term.

One of the problems that almost all the hypotheses generating approaches face is the large amount of spurious hypotheses generated in the process of automating the Swanson's ABC model. In order to eliminate the spurious hypotheses, different components of the biomedical ontology system, UMLS [12], have been utilized. Weeber et al. [13] used Metathesaurus of the UMLS to extract biomedical phrases and further limited the desired phrases by using the semantic types of the UMLS as an additional filter. Similar strategies are widely used by most of the follow-up research. Zhang et al. [3] and his colleagues used semantic network, another UMLS component that specifies possible relations among different semantic types, in order to restrict the association rules generated from the retrieval result set of topic A in the process of open discovery. Besides utilizing the biomedical ontology system, we envision that cross-repository validation may be another effective addition for eliminating spurious hypotheses.

No matter whether designed for closed discovery or open discovery, the described works are still constrained in the category of automating and refining Swanson's ABC hypothesizing model. Furthermore, all the approaches are based on retrieval result set of one or two initial topics provided by a user, instead of being able to scale up to the whole set of topics within a literature database for the purpose of discovering real, novel and cross-silo biomedical hypotheses.

If we model a biomedical literature repository as a comprehensive network of biomedical concepts belonging to different semantic types, the link discovery techniques may enable large-scale, cross-silo hypotheses discovery that goes beyond information retrieval-based discovery. Link discovery has been extensively studied on social networks such as Facebook, and bibliographic databases such as DBLP in recent years. As an important problem of link mining, link discovery refers to the discovery of future links between objects that are not directly connected in the current snapshot of a given network. In the following, we briefly review those link discovery techniques that are relevant to our work.

In the paper by Faloutsos et al. [14], the author proposed a measure called effective conductance to evaluate the goodness of a connection subgraph. Later, in the paper by Koren et al. [15], an improved measure called cycle free effective conductance was proposed by using only the cycle free paths in computing the proximity. This measure guaranteed that high degree intermediate nodes in the paths do not increase the proximity between two nodes unreasonably. The paper by Liben-Nowell and Kleinberg [16] discussed the problem of link prediction in social networks. It was one of the early works on link prediction that addressed the question of to what extent new collaborations (links) can be predicted by using the toplogy of the network. This work used an unsupervised approach to predict the links based on several network toplogy features in co-authorship networks. The paper by Al Hasan et al. [17] used a supervised learning approach for co-authorship link prediction based on simple neighborhood features, without factoring in any random walk features like effective conductance. Simple neighborhood features have several limitations compared to random walk features: they can not predict connecting paths of length greater than two (Benchettara et al. [18]), nor can they discriminate significant (good) paths from the set of all neighborhood nodes. The paper Benchettara et al. [18] used the bipartite nature of publication networks in a supervised learning framework. The paper Savas et al. [19] addressed the link discovery problem based on the number of paths of different lengths from multiple sources that exist between two nodes. However, this work did not factor in the different degrees of significances that different paths may have. Őzgür and his colleagues [9] applied link discovery technique to generate hypotheses on relationships between genes and vaccines. This work first extracted networks on gene-gene interactions and gene-vaccine interactions from literature with the help of gene and vaccine ontology; then analyzed the networks based upon different centrality measures calculated for each node in the networks. Given its limited focus on gene and vaccine relationships, this work by its nature was not designed for cross-silo biomedical discovery.

## Hypotheses generation as supervised link discovery on biomedical concept network

We model a biomedical literature as a concept network G, where each node represents a biomedical concept that belongs to certain semantic type, and each edge represents a relationship between two concepts. Each node or each edge is attached with a weight that reflects the significance of the node or the edge. In this work, we use the document frequency of a given node as its weight; use the co-occurrence of the two end nodes as the weight for the corresponding edge. Now, the hypotheses generation problem can be formulated as the process of link discovery on the concept network, i.e., the process of discovering all those pairs of nodes which are not directly connected in the current concept network but will be directly connected in the future. We further model the link discovery on the concept network as a process of supervised learning where a training data set is automatically generated from the concept network without class label assignments by domain subject experts. More specifically, we take two snapshots, namely $G_{t_{f}}$ and $G_{t_{s}}$, of the concept networks corresponding to two consecutive time durations t_*f *_and t_*s*_. That is t_*f *_is the first time duration and t_*s *_is the second time duration. We automatically collect a group of concept pairs that are not directly connected in $G_{t_{f}}$ and labeled each pair as either positive or negative. A concept pair is assigned the class label positive if this pair is directly connected in $G_{t_{s}}$; is assigned negative otherwise. For each collected pair, we further extract a set of features from $G_{t_{f}}$, such that a classification model can be built by using part of the labeled pairs as the training data. Once the classification model is learned, it can be used to predict the appearance of a new edge at a future time between two nodes that are not directly currently connected. The quality of the classification model surely depends on what features we can extract for the labeled pairs. Existing work in link discovery typically uses different types of topological features. We examine two types of topological features, namely random walk based and neighborhood based. Besides topological features, we also propose two semantically-enriched features, namely Semantic CFEC and Author List Jaccard. In the following, we will describe both topological and semantically-enriched features in detail.

### Topological features

Given a collected pair of nodes (s, t), we consider the following aspects of topology related to s and t: 1. the neighborhood of s and t; 2. the paths between s and t. To describe the neighborhood of s and t, the following measures are calculated:

• Common neighbors:

Score(s,t)=|τ(s)∩τ(t)|,

where *τ *(*s*) and *τ *(*t*) are the set of neighboring concepts for concepts s and t respectively.

• Adamic/Adar: The measure uses the common neighbors between two nodes and weights each of the common neighbors. It gives higher score for nodes with low degree.

$$Score\left(s,\;t\right)=\sum_{z\in \tau \left(s\right)\cap \tau \left(t\right)}\frac{1}{log|\tau \left(z\right)|}.$$

• Jaccard Co-efficient:

Score(s,t)=|τ(s)∩τ(t)|/|τ(s)∪τ(t)|.

• Preferential Attachment:

Score(s,t)=|τ(s)|⋅|τ(t)|.

To describe the paths between s and t, we examine the following features.

• Number of paths: more paths between s and t, more likely a future edge between s and t.

• Distance between s and t: longer it takes to reach s from t, less likely a future edge between s and t.

Given a pair of collected nodes (s, t), *the Cycle Free Effective Conductance (CFEC) *measure proposed in [15] can be used to describe the effects of both these two features on s and t on the likelihood of a future edge between s and t. We briefly explain the definition of CFEC below. The cycle-free escape probability (Pcf.esc(s→t)) from s to t is the probability that a random walk originating at s will reach t without visiting any node more than once. Let R be the set of simple paths from s to t (simple paths are those that never visit the same node twice). Cycle-free escape probability (Pcf.esc(s→t)) is defined using the following equation

$$Pcf.esc\left(s\to t\right)=\sum_{r\in R}Prob\left(R\right)$$

Cycle free effective conductance measure, is defined with the following equation:

$$\text{E}{\text{C}}_{cf}\left(s,\;t\right)=\deg_{s}\cdot {\text{P}}_{cf.esc}\left(s\to t\right).$$

From the above equation, it is clear that having multiple paths between two nodes will boost the score and thus addresses the first desired property. The definition also makes sure that already known information has no contribution to the score as it avoids cycles. In the random walk, a probability of transition from node i to node j is $p_{ij}=\frac{{\text{w}}_{ij}}{\deg_{i}}$. Thus, given a path P = v_1_, v_2_, . . . v_*r *_the probability that a random walk starting at v_1 _will follow this path is given by:

$$\text{Prob(}P\text{)}=\prod_{i=1}^{N}\;\frac{w_{v_{i}{v_{i}}_{+1}}}{\deg_{v_{i}}}$$

From the above equation it is evident that shorter paths are preferred.

### Semantically-enriched features

The above measures only evaluate network topology related features. However, each node that represents a biomedical concept is actually associated with rich semantic information. In this work, we consider the following two types of semantic information for a given node, its semantic type and its related author information.

**Figure 1:**
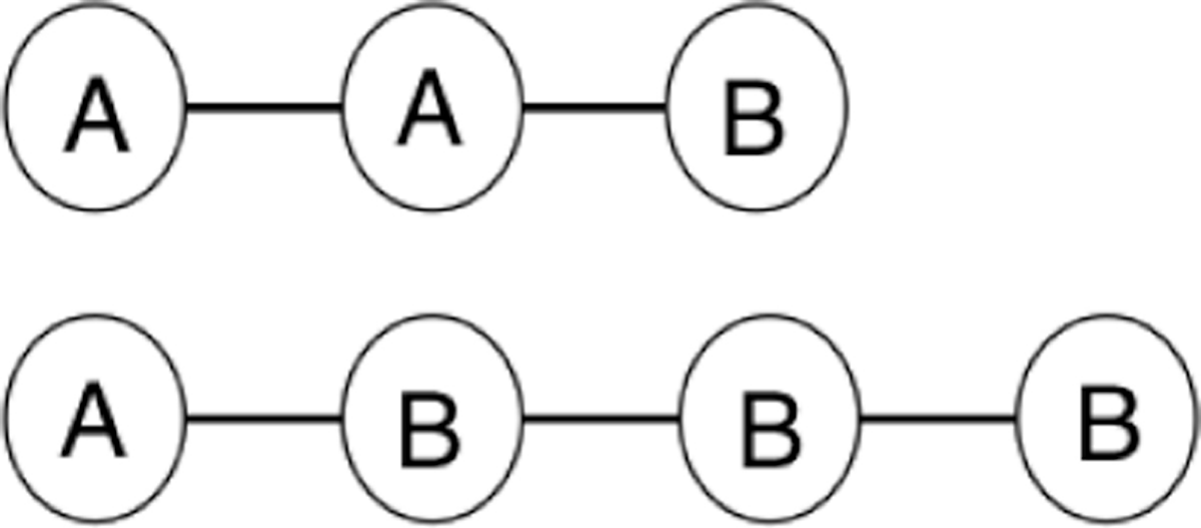
**Semantic simple paths**.

To factor in the semantic type of a given node, we propose a semantically-enriched CFEC measure that is called *Semantic CFEC*. The intuition behind using the semantic types of the intermediate nodes in a path is that connections formed between homogeneous nodes are less likely to be spurious connections. This observation has also been substantiated in the prior work of biomedical literature mining. The works by Weeber et al. [13] and Zhang et al. [3] used the UMLS semantic types to restrict the association rules or the hypotheses. Our proposed semantic-CFEC considers a subset of the simple paths, where each path has only those intermediate nodes whose semantic type is same as either the source node or the destination node. Let R* be the set of such simple paths called as semantic simple paths. *Semantic CFEC *is then computed using the paths r ∈ R*. Figure 1 shows some examples of such paths. To factor in the related author information for a given node, we propose another new measure that is called Author-List Jaccard. The intuition behind this measure is that two distant concepts may get connected due to the presence of enough researchers who are familiar with both the concepts. Let *author(s) *and *author(t) *be the list of authors who have published documents containing concepts s, t respectively. Then, we define this measure as below:

Score(s,t)=|author(s)∩author(t)|/|author(s)∪author(t)|

## Concept network creation and feature extraction using Map-Reduce framework

In this section, we describe the implementation of the computational model presented in the Hypotheses generation as supervised link discovery on biomedical concept network section. The major challenge to implement such a computational model is related to the need to process a huge amount of data. We use the Map-Reduce framework to implement the following three major components: 1) Extract a comprehensive biomedical concept network from the abstracts of all Medline papers published within 1990-2010; 2) Generate labeled pairs from two consecutive snapshots of the concept network; and 3) For each labeled concept pair, extract all the set of features described in the subsections titled Topological features and Semantically-enriched features.

### Concept network extraction

Each node of the concept network represents a biomedical concept, which is also attached with the following information: semantic type, related authors, and document frequency. Each edge of the concept network represents co-occurrence of the two end nodes in same documents. An edge is attached with the following information: the strength of the edge (i.e., the frequency of co-occurrence of the two end nodes), and the duration of the edge. The concept network is stored by using the following data structures.

• Concept-Document Map (CDM): The key of an entry in this map is a concept 'c' and year 'y', and the value of an entry is a set of document ids (PMIDS), where PMID is the ID of the Medline paper that concept *c *appears and year represents the publication year of this paper. Given a time duration *t*, we can easily derive a snapshot of CDM for t, denoted as *CDM_t_*, by taking a union of all the PMIDs for the keys 〈c, y〉, where the year 'y' is within the given time duration *t*. To generate this map in Map-Reduce framework each of the mappers processes a subset of the document collection and sends the tuple 〈concept, year〉 as the key and document list as the value to reducers. Reducers aggregate the document set for a given concept and year.

• Concept-Concept Matrix (CCM): We compute concept-concept associations from the set of concepts extracted from a PMID. That is, for each concept, we compute the co-occurring concepts within the same document. For each concept-concept association, we compute the co-occurrence frequency occurred in each year. Algorithm 1 describes the implementation of CCM in Map-Reduce framework.

• Concept-semantic Type: We extract the semantic type from UMLS Metathesaurus for each of the concepts.

• Concept-Author Map (CAM): The key of an entry in this map is a concept 'c' and year 'y', and the value of an entry is a set of authors. This map provides the set of authors who have published a document containing the given concept 'c' in a given year 'y'. Given a time duration *t*, we can easily derive a snapshot of CAM for *t*, denoted as *CAM_t_*, by taking a union of all the authors for the keys 〈c, y〉, where the year 'y' is within the given time duration *t*. To generate this map in Map-Reduce framework each of the mappers processes a subset of the document collection and sends the tuple 〈concept, year〉 as the key and author set as the value to reducers. Reducers aggregate the author set for a given concept and year.

**Algorithm 1: **Generating concept-concept matrix

**Data**: Document Corpus

**Result**: Concept-Concept Matrix

initialization *CCMis_local matrix*;

Map:

**for ***each mapper m ***do**

**for ***each document d_i _in document corpus*

*of mapper m ***do**

**c(i) ← **set of concepts extracted from *d_i_*;

*y_i _*← published year of *d_i_*;

**for ***each concept pairs c_k_, c_l _of c(i) ***do**

*CCM_local[c_k_, c_l_, y_i_] *← *CCM_local[c_k_, c_l_, y_i_] *+ 1;

end

end

**for ***each entry (c_k_, c_l_, y_i_) in CCM_local ***do**

key ← *(c_k_, c_l_, y_i_) *;

value ← *CCM_local(c_k_, c_l_, y_i_) *;

*return (key, value)*;

end

end

Reduce

**for ***each key (c_k_, c_l_, y_i_) and *$count_{1}^{n}$**do**

$\text{sum}\leftarrow \sum_{i=1}^{n}{count}_{i};$

*CCM(c_k_, c_l_, y_i_) *← sum;

end

Given a comprehensive concept network stored in the above data structures, we apply Algorithm 2 to derive a snapshot of the concept network for a given time duration *t *in Map-Reduce framework. A snapshot of the concept network is stored in a graph data structure.

### Automatic generation of class labels for concept pairs

Given two snapshots $G_{t_{f}}$ and $G_{t_{s}}$ of the concept network corresponding to two consecutive time duration *t_f _*and *t_s_*, we generate a group of labeled pairs based on which a training data set can be formed for the proposed supervised link discovery. The following process describes how we automatically assign class labels to concept pairs without any involvement of subject domain experts.

For a pair of nodes (i, j) that is not directly connected in $G_{t_{f}}$, we categorize its possible connection situations in $G_{t_{s}}$ as follows:

• Connection is strong in $G_{t_{s}}$: There is an edge between i and j in $G_{t_{s}}$, namely e_*ij *_, and we have e_*ij *_.*strength *≥ *min_support*.

• Connection is emerging in $G_{t_{s}}$: There is an edge between i and j in $G_{t_{s}}$, namely e_*ij *_, and we have *margin *× *min_support *≤ *e_ij_*.*strength *<*min_support*, where 0 <*margin *< 1.

**Algorithm 2: **Generating the snapshot of the concept network, G_*t*_, for a time duration t

**Data**: Concept-Concept Matrix CCM, Concept-Document Map CDM, time duration t

**Result**: Snapshot of the Concept Network for a time duration t

initialization Create CDM_*t*_: the snapshot of CDM for the time duration t;

**for ***each *〈*key(c_i_, c_j_, y_k_),value(val)*〉 *in CCM ***do**

**if ***y_k _*∈ *t ***then**

**if ***no node exists for c_i _***then**

create a node v_*i *_for c_*j *_; v_*i*_.name = c_*i*_;

v_*i*_.frequency = (CDM_*t*_.get(c_*i*_)).size();

end

**if ***no node exists for c_j _***then**

create a node v_*j *_for c_*j *_; v_*j*_.name = c_*j*_;

v_*j*_.frequency = (CDM_*t*_.get(c_*j*_)).size();

end

**if ***no edge links for *〈*v_i_, v_j_*〉 **then**

create an edge e_*ij *_between v_*i *_and v_*j*_

e_*ij*_.strength = 0;

end

e_*ij*_.strength = e_*ij*_.strength + val

end

end

• Connection is weak in $G_{t_{s}}$: There is an edge between i and j in $G_{t_{s}}$, namely e_*ij*_, *e_ij_*.*strength *<*margin *× *min_support*, where 0 <*margin *< 1.

• No direct connection in $G_{t_{s}}$: There is no edge between i and j in $G_{t_{s}}$.

Given a pair of nodes that has no direct connection in $G_{t_{f}}$, we assign the class label positive to it if this pair's connection is strong in $G_{t_{s}}$; assign the class label negative to it if this pair's connection is weak in $G_{t_{s}}$ or there is no direct connection in $G_{t_{s}}$. If this pair's connection in $G_{t_{s}}$ is emerging, its class label should be emerging, however, we don't consider this class in this work. The major challenging issue of generating labeled pairs is that there would be a huge number of pairs that are not directly connected in $G_{t_{f}}$. In order to address this issue, we use the following procedure to generate labeled pairs.

• For each pair whose connection is strong in $G_{t_{s}}$, if it has no direct connection in $G_{t_{f}}$, assign positive to this pair.

• For each pair whose connection is weak in $G_{t_{s}}$, if it has no direct connection in $G_{t_{f}}$, assign negative to this pair.

• Select a random sample of the nodes in $G_{t_{f}}$ and generate concept pairs from the selected random sample. If a pair has no connection in both $G_{t_{f}}$ and $G_{t_{s}}$, assign negative to it.

The number of labeled pairs generated from a large-scale concept pairs can be huge. Furthermore, the number of positive pairs and negative pairs can be highly unbalanced. To address these issues, we randomly select certain portion of positive and negative pairs to form a training data set.

### Feature extraction

For each of labeled concepts pair, we extract all the set of features described in the subsections titled Topological features and Semantically-enriched features from the snapshot of the concept network $G_{t_{f}}$. Given the fact that the number of labeled pairs is large, feature extraction is also a computationally expensive step. To address this problem, the feature extraction is implemented on a map-reduce framework. The distributed implementation of feature extraction can be described in the following way:

1. Trim $G_{t_{f}}$ such that it only contains edges with strength greater than or equal to the minimum support. Store the trimmed $G_{t_{f}}$ in each of the mapper's main memory. After trimming, $G_{t_{f}}$ is much smaller, so it is feasible to store it in memory.

2. Distribute the labeled pairs among the mappers. Each mapper extracts the features for a subset of concept pairs using the trimmed $G_{t_{f}}$.

## Experimental results

We study the following aspects of our proposed methodology in our experimental set-up:

1. The performance of the proposed supervised link discovery approach. More specifically, we evaluate whether the proposed approach is able to conduct reasonable predictions on concept links that are currently weak or non-existing but may become strong in the future. Since predictions are carried out based on a classification model that is built upon a training data set extracted from two consecutive snapshots of the concept network, the performance of link discovery can be evaluated by measures such as classification accuracy, recall, and precision as results of n-fold cross validation on the training data.

2. The effect of the parameters min-support and margin on the performance of link discovery. These two parameters are used in generating class labels for concept pairs of the training data.

3. The effect of the proposed features for each concept pair, such as CFEC, Semantic-CFEC and Author-Jaccard, on the performance of link discovery.

4. The effect of using different snapshots of the concept network to generate training data. For this purpose, we first take three consecutive snapshots of the concept network, each of which spans a 5-year period; then generate the first training data set from the first two snapshots and the second training data set from the last two snapshots. Accordingly, we compare the performance of classification models built on these two training sets.

5. The effects of different supervised learning methods on the performance of link discovery. For this purpose, we experiment with two typical supervised learning methods, one is C4.5 decision tree and the other is Support Vector Machine(SVM). Decision tree generates results that are easy to interpret, whereas SVM is well received due to its outstanding performance in various applications.

### Experimental setting

We processed the MEDLINE records from 1990-2010 to build the base concept network. From each of the MEDLINE record, which is a XML file, we extract the following information to build the concept network: Authors, Dates, Document ID (PMID), Keywords from fields such as MeshHeadingList, Chemical Compounds List and Gene Symbol List. Table 1 shows some important statistics of the generated concept network.

**Table 1:** Statistics of the generated concept network

Total number of concept pairs	17356486
Total number of documents	11021605
Total number of concepts	165674

**Figure 2:**
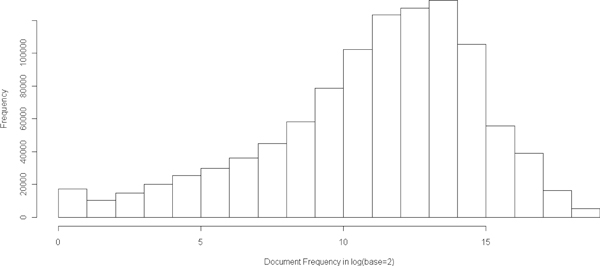
**Histogram of concept document frequency**.

**Figure 3:**
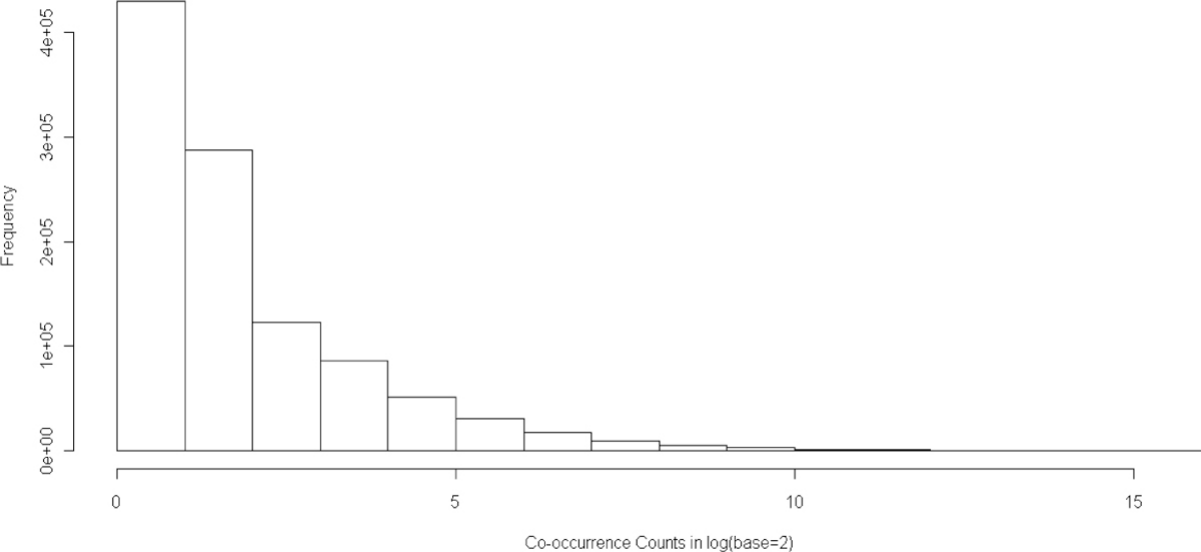
**Histgram of co-occurence counts**.

**Figure 4:**
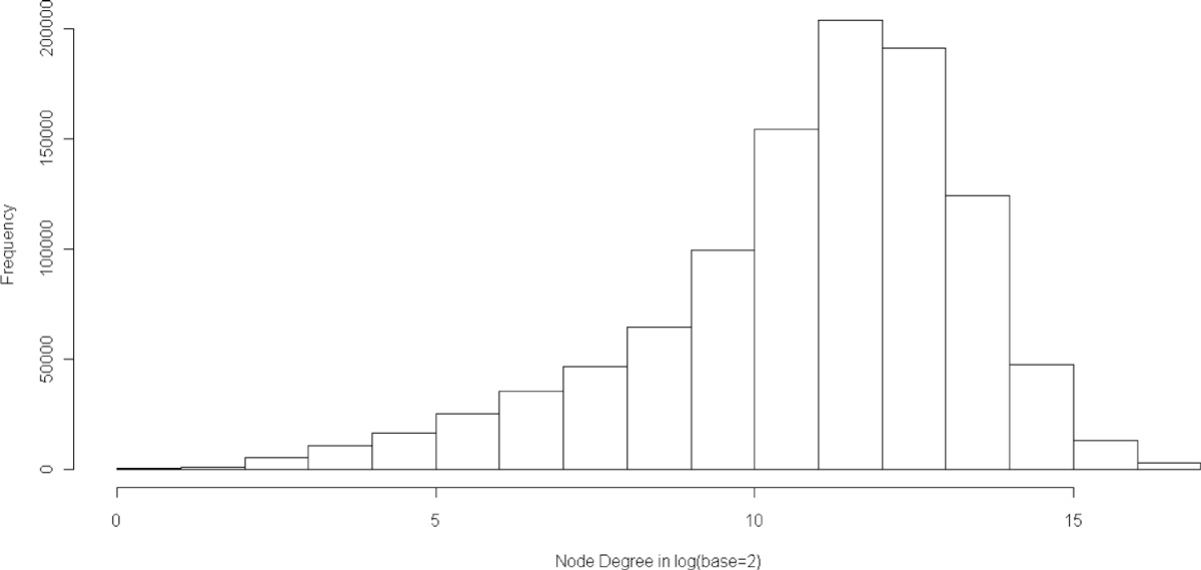
**Histogram of degree of the nodes**.

We further show the distribution of document frequency of concepts in Figure 2, the distribution of co-occurrence frequency of concepts in Figure 3, and the distribution of degree of concept nodes in Figure 4. From these distributions, we observed that 1) majority of the concepts have document frequency greater than 1000; 2) majority of the concepts link to at least 1000 other concepts; and 3) among all linked concept pairs, around 33% have co-occurance frequency greater than 4 and around 20% have co-occurance frequency greater than 8.

Based on the concept network, the following snapshots were generated: $G_{t_{1}}=1991-1995$, $G_{t_{2}}=1996-2000$ and $G_{t_{3}}=2001-2005$. We generated the first set of labeled pairs from $G_{t_{1}}$ and $G_{t_{2}}$. As shown in Table 2, the number of labeled pairs, especially the number of negative instances, is too large for a typical supervised learning algorithm. Therefore, we randomly select 10% of positive instances and 10% of negative instances from the first set of labeled pairs generated from $G_{t_{1}}$ and $G_{t_{2}}$ to form the first training data set. For each labeled pair in the first training data set, we extracted its features solely from $G_{t_{1}}$. Then we generated the second set of labeled pairs from $G_{t_{2}}$ and $G_{t_{3}}$. By taking 10% of positive instances and 10% of negative instances from the second set of labeled pairs, we form the second training data set. For each labeled pair in the second training data set, we extracted its features solely from $G_{t_{2}}$.

**Table 2:** Number of instances

	Test support value			
	
	4	6	8	10
+Ve instances	597167	460230	233276	154509
-Ve instances	5752307	7390843	7734204	7734204
Emerging	5404526	5730614	5614207	5692974

We first applied C4.5 Decision Tree on the training data set generated from $G_{t_{1}}$ and $G_{t_{2}}$ to study the effects of parameters and proposed features on the performance of the proposed approach; then studied the performance of C4.5 Decision Tree built on both training data sets; finally compared the performance of C4.5 Decision tree and SVM based on both training data sets. A 10-fold cross validation was used to evaluate classification accuracy, recall, precision and F-Measure in all experiments.

### Support and margin

We generated the labeled pairs by using the procedure described in the Automatic generation of class labels for concept pairs section with different values for the variable *min_support *and for the variable margin. The number of positive instances and the negative instances generated for training purpose is highly unbalanced. Table 2 shows the number of positive and negative examples for different values of *min_support*. Given the fact that unbalanced data sets are difficult to train on, we performed an under-sampling of the majority class.

**Figure 5:**
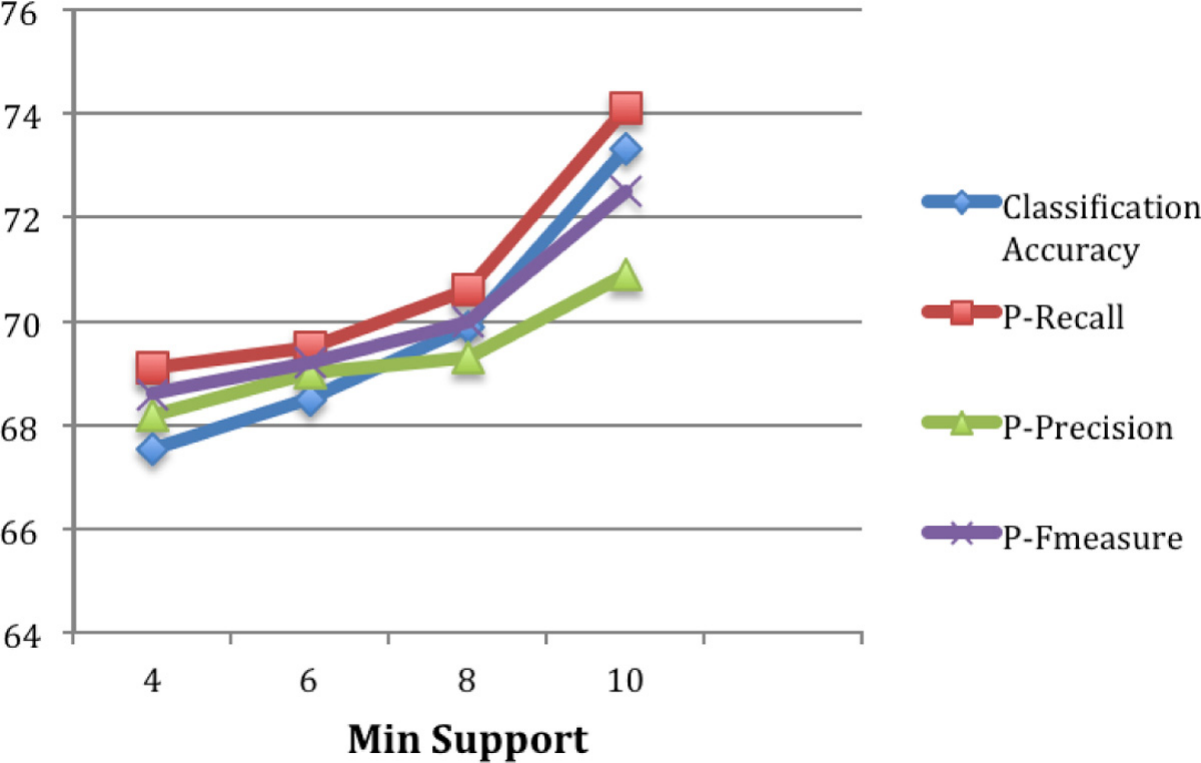
**Varying minimum support for test duration vs model performance**.

Figure 5 shows the classification results obtained on the test data set by varying the value of *min_support *from 4 to 10 for a fixed value of 0.3 for the variable *margin*. We present classification accuracy, recall for the positive class (P-Recall), precision for the positive class (P-Precision) and the F-Measure for the positive class (P-Fmeasure). As can be seen from Figure 5, the model accuracy in terms of all 4 measures increased as we increase the value of *min_support *from 4 to 10. The classification accuracy increased from 67.5% to 73.4% as the *min_support *is increased from 4 to 10. The explanation for the improvement in the model accuracy is as follows: As we increase the value of min support, some of the labeled pairs which are considered to be strong connections at a lower value will no longer be strong connections at a higher value, but will fall into the category of emerging connections. This means, our feature set has a better discriminating ability to choose between the strong connections and weak connections as compared to that of emerging connections and weak connections.

**Figure 6:**
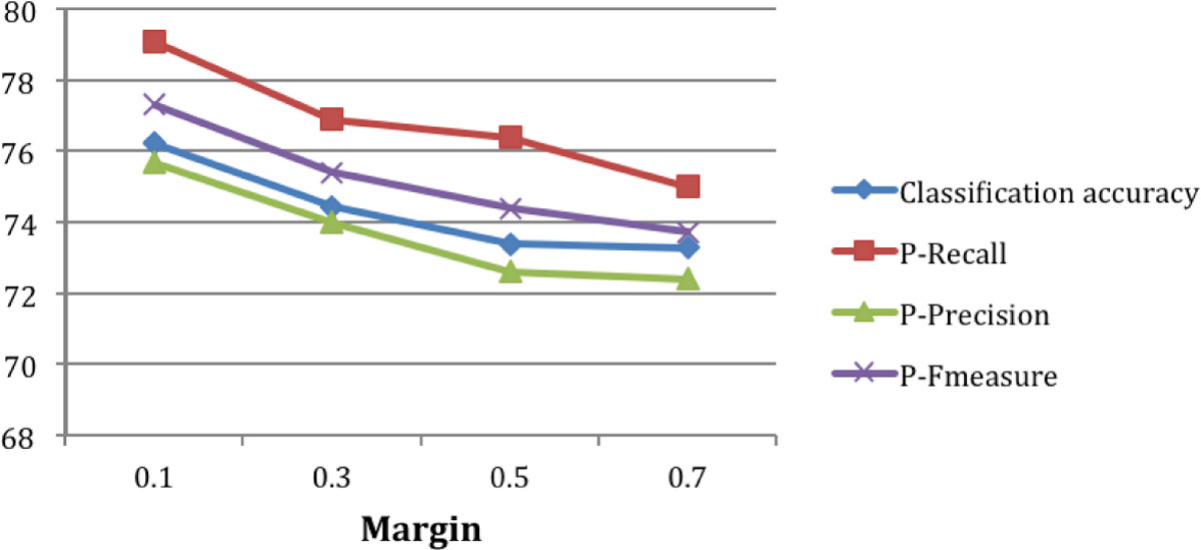
**Varying values of margin**.

We have also experimented with different values for the variable *margin*. Figure 6 illustrates the results of the classifier as increase the value of *margin *from 0.1 to 0.7. The best results are obtained with *margin *0.1. We obtained a classification accuracy of 76.2% with *margin *0.1. As the margin increases, there will be more negative examples and the data becomes even more unbalanced.

### Semantically-enriched features

**Figure 7:**
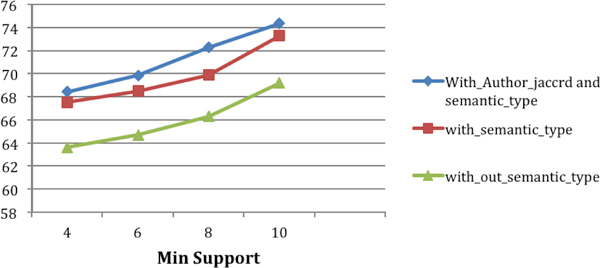
**Classification accuracy for feature sets**. (a) Without semantic type and author jaccard features. (b) With semantic type features but no author jaccard feature. (c) All features.

We proposed two semantically-enriched features, Author_List Jaccard, and Semantic CFEC. Figure 7 illustrates the usefulness of Author_List Jaccard towards the improvement in the classification model. Figure 7 also illustrates the improvement that we obtained by adding Semantic CFEC. Figure 7 also shows the relative improvements that were obtained by adding the features Author_List Jaccard and Semantic CFEC. The feature Semantic CFEC improved the classification accuracy by 6% and the feature Author_List Jaccard improved the classification accuracy by another 2%.

### Two different training data sets

**Figure 8:**
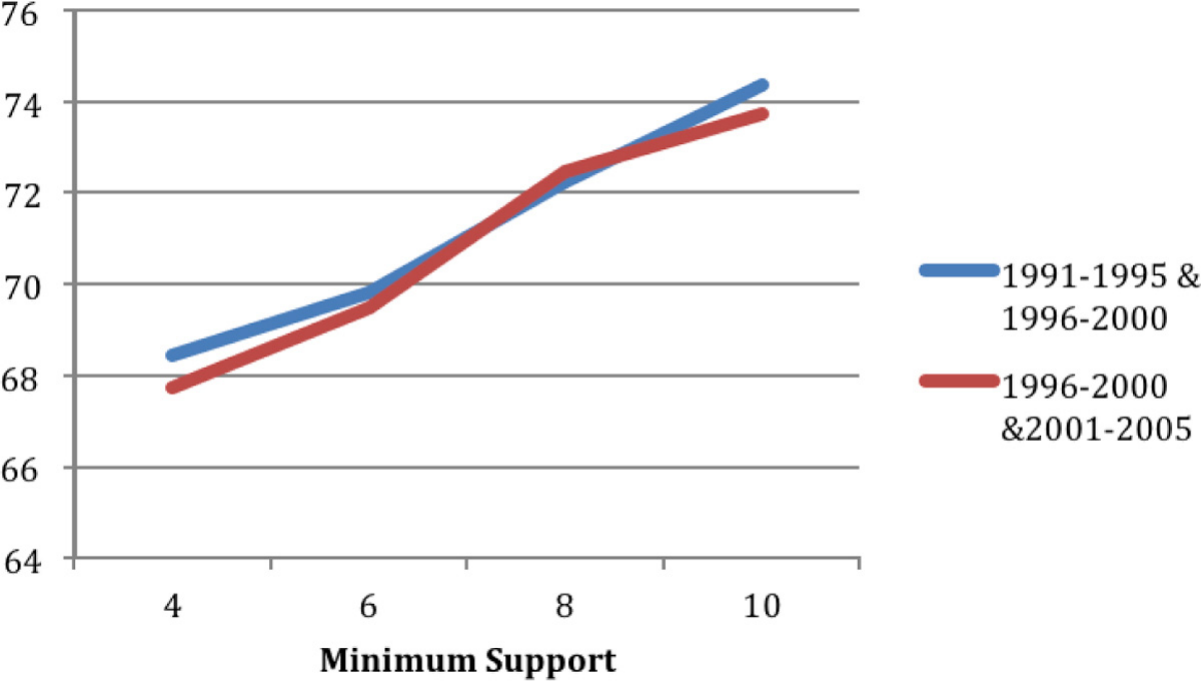
**Comparison of classification accuracy for two different training data sets (training set 1 was extracted from concept network snapshots $G_{t_{1}}=1991-1995$ and $G_{t_{2}}=1996-2000$; training data set 2 was extracted from concept network snapshots $G_{t_{2}}$ and $G_{t_{3}}=2001-2005$)**.

In Figure 8, we compare the classification accuracies corresponding to two different training data sets. Recall that the first training data set was extracted from concept network snapshots G_*t*1 _and G_*t*2_; whereas the second training data set was extracted from snapshots G_*t*2 _and G_*t*3_. As can be seen from the figure, the classification accuracies are consistent across two different training data sets.

### C4.5 decision tree vs. SVM

**Figure 9:**
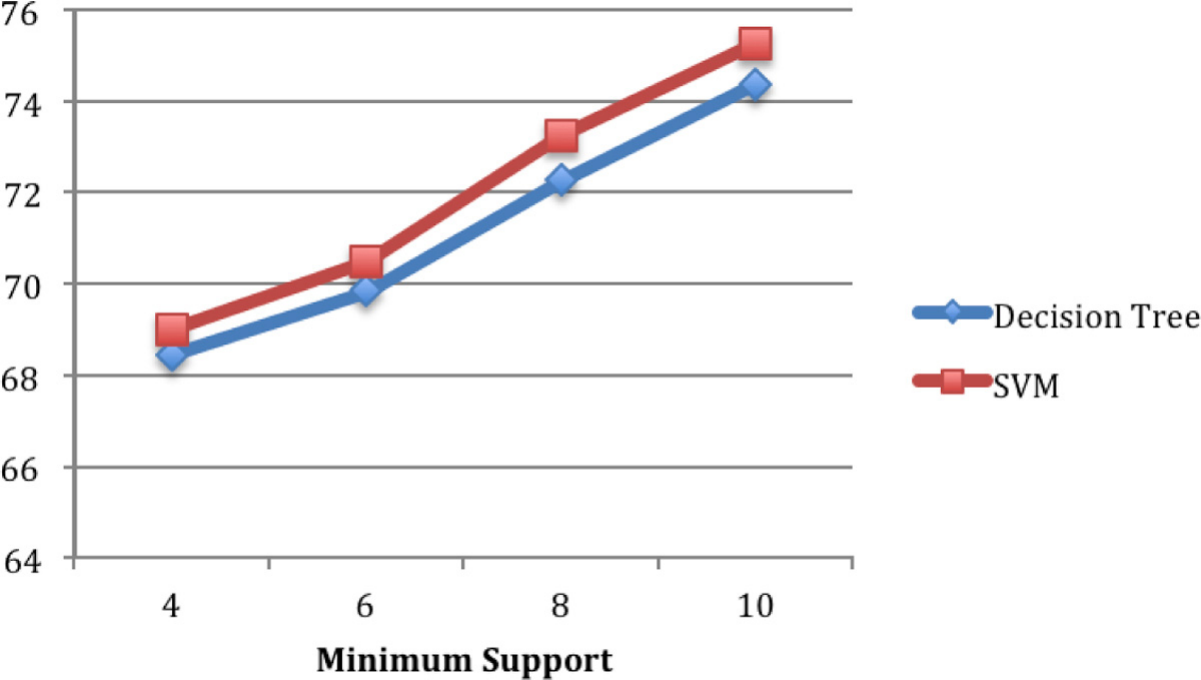
**Classification accuracy using SVM and decision tree on training data set 1 (extracted from snapshots $G_{t_{1}}=1991-1995$ and $G_{t_{2}}=1996-2000$)**.

**Figure 10:**
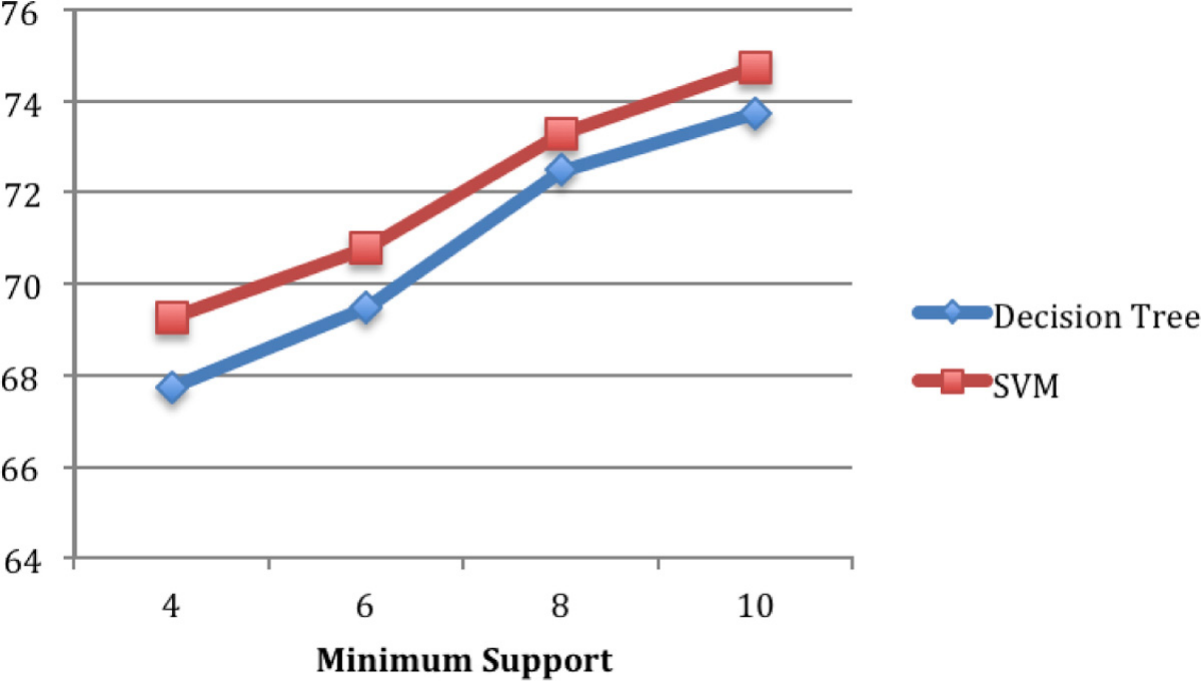
**Classification accuracy using SVM and decision tree on training data set 2 (extracted from snapshots $G_{t_{2}}=1996-2000$ and $G_{t_{3}}=2001-2005$)**.

Figure 9 illustrates the comparison of the classification accuracy obtained using SVM and C4.5 decision tree on the first training data set that was extracted from concept network snapshots G_*t*1 _and G_*t*2_. We used radial basis function (RBF) as the kernel type for SVM. Libsvm [20] is used as the SVM library. The results from SVM are slightly better (1% to 2%). In Figure 10, we show the similar result of comparison for the second training data set that was extracted from concept network snapshots G_*t*2 _and G_*t*3_.

### A case study

If we consider the time duration from 1991 to 1995, there exists no Medline record in this time duration that mentioned both of "Prostatic Neoplasms" and "NF-*κ*B inhibitor alpha". Document frequency of "Prostatic Neoplasms" in this time duration is 6807, whereas document frequency of "NF-*κ*B inhibitor alpha" is 91. However, the co-occurence frequencies of this concept pair are 15 and 42 corresponding to the MEDLINE corpus in the time durations 1996-2000 and 2001-2005, respectively. It is worthwhile to study if the supervised learning model built from the first training data set is able to predict the strong connection between these two concepts after 1995.

Recall that, in our experimental study, the first training data set was formed by randomly selecting 10% of labeled pairs generated from concept network snapshots G_*t*1 _= 1991-1995 and G_*t*2 _= 1996-2000. We first made sure that the pair "Prostatic Neoplasms" and "NF-*κ*B inhibitor alpha" is not part of the first training data set. Then we run the supervised learning model built on the first training data set to make a prediction for this pair. The model successfully predicted the strong connection between these two concepts after 1995 by assigning a positive class label to this pair.

Furthermore, we extracted the paths between these two concepts, which may provide clues on why these two concepts may potentially link to each other. Table 3 shows the six most significant paths using Cycle Free Effective Conductance (CFEC) feature to sort the paths connecting the given concepts.

**Table 3:** Significant paths using cycle free effective conductance feature

Prostatic Neoplasms →	Tumor Necrosis Factor-alpha →	NF-*κ*B inhibitor alpha
Prostatic Neoplasms →	RNA, Messenger →	NF-*κ*B inhibitor alpha
Prostatic Neoplasms →	Adenosine Triphosphate →	NF-*κ*B inhibitor alpha
Prostatic Neoplasms →	Oligopeptides →	NF-*κ*B inhibitor alpha
Prostatic Neoplasms →	Tetradecanoylphorbol Acetate →	NF-*κ*B inhibitor alpha
Prostatic Neoplasms →	Cycloheximide →	NF-*κ*B inhibitor alpha

## Conclusions

Modeling a biomedical literature repository as a comprehensive network of biomedical concepts and viewing hypotheses generation as a process of automated link discovery on the concept network representing the literature repository, opens the door for performing large-scale cross-silo biomedical hypotheses discovery. We have presented the methods to generate a concept network and concept-author map from large-scale literature repositories using Map-Reduce framework. The link discovery on the concept network was further modeled as a classification problem and we proposed a framework to automatically generate the labeled instances of concept pairs for supervised link discovery. Our method also extracts multiple heterogeneous features for labeled concept pairs. These features include path based features such as *cycle free effective conductance (CFEC)*, neighborhood features such as preferential attachment. In addition, we proposed a new feature based on CFEC namely *semantic-CFEC*, which utilizes the semantic type of the nodes in the path. Another important contribution of work is the use of author information. To the best of our knowledge, this is the first work that exploited the connecting two concepts via author links associated with those concepts for hypotheses discovery. Through experimental results, we showed an improvement of 7-9% in classification accuracy of link discovery obtained due to the addition of semantic type and author based features.

As part of the future work, we will explore using ensemble methods such as gradient descent boosted decision trees for classification. We will also explore the prediction of emerging connections between concepts in addition to the prediction of strong connections. A web service that generates biomedical hypotheses based on the proposed method will be built and published.

## Competing interests

The authors declare that they have no competing interests.

## Authors' contributions

JRK researched the area of link discovery methods and proposed the supervised link discovery method for biomedical hypotheses discovery. YX and VVR proposed further improvements to the methodology. JRK implemented the proposed method and generated experimental results. AG generated the input data sets and also formatted the manuscript. YX and VVR organized the manuscript in a formal way. All authors have contributed to the writing of the manuscript.
